# 

**Published:** 1812-06

**Authors:** 


### MONTHLY CATALOGUE OF MEDICAL BOOKS.

PHARMACOLOGIA, or the History of Medical Substances, in order to enable the practitioner to prescribe them with efficacy and elegance, and to dispense them with accuracy. By John Ayrton Paris, M.B. F.L.S. 12mo.—Callow.

Correct Synopsis of the London Pharmacopœias of 1787 and 1809; exhibiting, in the first column, an alphabetical list of articles changed either in name or in preparation, and in the second column their synonymes, if any.—Cox.

---

Mr. Edward Leese, Member of the Royal College of Surgeons, has in the press, an Explanation of the Causes why Vaccination has sometimes failed to prevent Small-pox; and also, a description of a method, confirmed by experience, of obviating such causes.

Mr. Thomas Foster, F.L.S. has in the press, Physiological Reflections upon the dangerous effects of Spirituous and Fermented Liquors.

---

### *NOTICES TO CORRESPONDENTS.*

*We acknowledge with pleasure the receipt of Communications from Dr. Spalding (Portsmouth, New Hampshire); Dr. J. S. Blount (Paris); Mr. F. (Jamaica); T. E. B.; &c.*

*Authors who are desirous that their Works should meet with early notice in this Journal, are requested to direct their publisher to forward them, addressed to the Editors, at No. 47, Ludgate-hill.*

*Sir John Sinclair's Letter on some remedies for Calculus, did not come to hand in time for this Number, but will certainly have a place in our next.*

---

### *CORRIGENDA.*

Page 374, line 20, for *of it,* read *of the cartilage.*

### INDEX.

ALMONDS, essential oil of, experiments with 2, 6
Animals, living, remarks on experiments upon 7
Animal heat, Mr. Brodie's experiments on its cause 8
Atmosphere, observations on 17
Aneurism, popliteal operation for 29
Animal food, reasons for eating 47
Apothecaries, on the remuneration of 52, 53
Animal and vegetable substances, art of preserving 75
Abscess of the liver, case of, by Dr. Paxton 95
Apothecaries and physicians in partnership 116
Accoucheur physicians, remarks on 117
Acid, camphoric, experiments on 127
Atmosphere, syphilitic 138
Apoplexy, different species of, Dr. Montain on 151
Anderson, Dr. on an affection of the stomach from external use of muriate of mercury 167
Atmosphere, state of, and diseases, their connection 264
Adams, Dr. Jos. essay on croup 333
Amputation at the shoulder joint, success of 378
Animalcula, diseases occasioned by 379
Acid, Prussic, experiments on 404
Arnica montana, changes produced on by the eggs and larvæ of insects 406
Acid, oxygenated-muriatic, anti-contagious properties of 408
Ague, jumping, case of, by Lord Monboddo 486
Anatomy, morbid, treatise on 494
Alimentary canal, anatomy of its diseases 497
Alvine concretions, history of 501
Brodie, Mr. experiments with vegetable poisons 3
——— to ascertain the cause of animal heat 8
——— on the influence of the brain upon the heart ib.
Beauvois, M. de P. Flora of Benin and Oware 22
BOOKS, NEW MEDICAL, &c. CRITICAL ANALYSIS OF.—An Inquiry into the Process of Nature in repairing Injuries of the Intestines, &c. by B. Travers, 55-128. Some Observations upon Diseases, chiefly as they occur in Sicily; by W. Irvine, M.D. 65. Practical Observations on Cancer; by the late John Howard, 67. German Syphilitic Physician; by S. C. Meyer, M.D. 137. Diseases of the Generative System; by J. Robertson, 139. Journal General de Medecine, &c. 151. Hortus Botanicus Americanus; by N. T. Titford, M.D. 159. Edin. Med. and Surg. Journal, No. 28. 159. Saunders and Farre on Diseases of the Eye, 229, 335. Gillman on the Bite of a Rabid Animal, 236. Newton's return to Nature, 312. Muter's Observations on operating for Cataract, 318. Edinburgh Journal, 323. Balfour's Sol-lunar Influence, 398. Annales de Chimie, 403. Journal General de Medecine, 410. White on contracted Intestinum Rectum, 413. Tupper on Sensation in Vegetables, 417. Edinburgh Journal, 418. Account of Proceedings for establishing Sea-water Baths, 423. B. Clarke's Experiments on the Foot of the Horse, 425. Dr. Monro's Morbid Anatomy, 494. Crowther on Insanity, 513. Bullock's Companion to the London Museum, 516.
Brazil salt, and of its manufacture 76
Baths of sea-water, intended construction of in London 77, 342
Botanical report 82, 258, 347, 524
Blood, Brande, on 73
Brown, Dr. on fistula in ano 75
Brande, Mr. experiments to prove the non-existence of iron in the blood 73
Blake, Mr. on hooping cough 102
Bath, vapour, queries on 95
Bartlet, Sophia, case of S. P. a second time 110
Bronchocele, inquiry on its cure 114
Boorde, Andrew, Breviarie of Health 116
Bruyn, de, his secret, what 118
Buxton, Dr. cases of phthysis 159
Bryce, Mr. effects on the arm by variolous inoculation after vaccination 165
Bradley, Dr. observations on puerperal fever 183
Bleeding, its use in intermittents 190
Bougie, its use in cure of procidentia ani 269
Bile, an excretory fluid 280
Boy, idiot, his remarkable attachment to bees 310

Bell, John, arguments against the wind of a ball 326
Bullock, Mr. museum of nat. hist. extensive collection and arrangement of 341
Bladder, muscular coat of, observations on, by Dr. Hunter 381
Barton, Prof. observations on vegetable muscicapæ 388
Brodie, Mr. further experiments on the action of poisons 427
Bone-set, (*Eupatorium perfoliatum,*) observations on 428
Brown, Mr. instrument for compressing the subclavian artery 430
Blisters, on preventing strangury from 453
Bleeding, danger of neglecting in acute diseases 481
————, conclusions respecting, in inflammation 483
Birds; genus Paradisea, Cuculus, and Trochilus, in London Museum, observations on 517
Cardamom of Malabar, account of 19
Cuvier, M. on fossil remains 23
Collis's, Mr. surgical anatomy 29
Corps Charlatanique 31
Chrestien, Dr. preparation of gold 32
Cynanche trachealis, on 45
Catalogue, monthly, of medical books 88, 176, 264, 352, 440, 528
Cases—Of psora, 45; pneumonia treated by copious bleeding, 94; abscess of the liver, 95; scarlatina, 105, 6, 7, 8; of the effect of variolous fluid on the arm after vaccination, 165; of abortion from diseased placenta, 168; of vaccine and chicken pox occurring together, 171; of paralysis, 149; of pulmonary consumption, 159; of strangulated hernia, 162; of popliteal aneurism, 177; of sudden death in the last month of pregnancy, 179; of visceral inflammation with dissection, 189; of pneumonic inflammation, 198; of croup, 213; of trance, 227; of rabies in a dog, 241; of rabies in swine, 243; of rabies in a boy, 253; of gun-shot wound, 265; of procidentia ani, 269; of ptyalism, 277; abscess of the liver, 278; of tic douloureux, 281; of cynanche trachealis, 282; rheumatism acute, 287, 290; Cæsarean operation, 330; diabetes relieved by bleeding, 333; removal of metacarpal bone, ib.; of wounded diaphragm, ib.; puerperal convulsions, 345; of croup, 365; periodical hæmorrhoidal discharge, 410; of torpor with dissection, 418; of tying the iliac artery, 419; of erythema mercuriale, 420; of chorea, 421; exemplifying the effects of friction, 421; of small-pox after vaccination, 422; bite of the coluber naga, 432; ascites cured by tapping through the vagina, 434; fracture of the skull, 435; stramonium in asthma, 436; femoral hernia, ib.; hydrophobia, 437; of lake fever cured by eupatorium perfoliatum, 449; of indigestible substances taken into the stomach, 453; of typhus cured by purging, 458; of lacerated wound, 464; of polypus protruded at the time of labor, 467; case of a marine, by Mr. Scott, 473; case of phrenetis, 477; of jumping ague, 486; of injury of the head, 521.
Collectanea medica, chemica, &c. &c. 115, 216, 295, 380, 485
Contagion, morbid, its permanence 118
Cantharides, experiments on, by Rubiquet 127
Camphoric acid, experiments on, by Bucholz 127
Chancre, remarks on 142
————, local treatment of 143
Cantharides, internal use of 145, 6
Couching-needle, use of in infants 161
Comet, new one 168
Cæsarean section, observations on, 181; case of 330
Croup, dissertation on, by Dr. Hosack 195
————, three stages 204
————, emetics serviceable in 206
Calomel, its efficacy in croup 209
Croup, Dr. Mitchell's own case of 213
Conjunctiva, inflammation of, in children 230
Comet, Herschel on 256
Catheter elastic gum, used to convey fluids into the stomach 269
*Cinchonin*, essay on 295
Cataract, congenital, observations on 335
Croup, essay on 353
————, contagion of 362
————, case of 365
Cancerous parts, structure of 366
Caterpillars, observations on the destruction occasioned by 376
Caustic, success of its application in tetanos 378
Custom, singular, of lying-in women 381
Cold, great degree of, borne by reptiles and insects 485

Concretions alvine, history of 501
Davy, Mr. on the combinations of oxymuriatic gas and oxygen 15
Dumas, M. observations sur la physiognomie propre a quelques malades chronique 38
Depletion by the lancet, Mr. Scott on 89, 472
Diseases of debility, treatment of 145
Duncan, Dr. report of the epidemics of Edinburgh in 1810 166
Diseases and states of the atmosphere, their connection 264
Denman, Dr. on the structure of cancerous parts 366
——— case of polypus 467
*Datura metel*, observations on 524
Ellis, Mr. observations on the atmosphere 17
Elettaria, account of 20
Eau medicinale, Moore's analysis of 33
Experience and experiment in medicine, their meaning explained 2
Electricity, its use in a case of paralysis 101
Edinburgh, report on the epidemics of, in 1810 166
Emetics, their utility in croup 206
Edmonston, Dr. natural and medical hist. of the Zetland sheep 216
Eyes, peculiar disease in those of the Zetland sheep 219
Eye-lid, inversion of the upper, its cure 235
Eye, illustration of its important changes of structure 235
———, on some peculiarities in the muscles of 257
Enemas of lemonade, remarkable instance of relieving thirst and febrile irritation 267
Ellis, Mr. observations on wind of a ball 323
*Eupatorium perfoliatum*, and *cannabinum*, remarks on their medicinal properties, 428; Mr. Pursh on 449
Electricity, medical, Mr. Phythian on 451
Fossil animal remains in Germany, France, England, and America 23, 28
Fever, yellow, Dr. Bancroft on 36
Fistula in ano cured by decoction of the flowers of the *prunus spinosa* 95
Facts, veracity in reporting 115
Forbes, Mr. on tropical nyctalopia 165
Fog, dense, and remarkable darkness in London 175
Fever, puerperal, observations on 183
*Floræ Borealis Americanæ*, prodromus of 344
Fogo, Mr. observations on twin cases 367
Fever, puerperal, memoir on 377
———, hospital, memoir on acetite of ammonia in 378
Fourcroy, instance of his ignorance of a fact 382
Geological Society of London, its transactions 28
Gold, Dr. Chrestien's preparation of 32
Generation, equivocal, on 50
Ginklofe (trismus nascentium) of Iceland, its history and fatality 123
Generation, diseases of the female organs of 141
Gold, memoir on some medicinal preparations of 156
Gibson, Mr. on the use of the couching-needle in infants 161
Geoghegan, Mr. on strangulated hernia 162
Gomes, M. B. A. essay on *cinchonin* 295
Ganoo of India (goitre of the Alps) observations on 382
Gibson, Mr. death, and short biography of 438
Green, Mr. case of lacerated wound 464
Hutchinson, Dr. on popliteal aneurism 29, 177
Howard, Mr. on cancer 67
Home, Mr. on the passage of fluids from the stomach into the circulation 13
———, on the fluid in the cells of the spleen ib.
Hamilton, Mr. on equivocal generation 50
Hooping cough, a remedy for 102
Holland, Dr. Henry, on the diseases of the Icelanders 118
Hypochondriasis, common in Iceland 123
Hernia, strangulated, cases of 162
Hosack, Dr. New York, dissertation on croup 195
———, on hives ib.
Hammick, Mr. case of gun-shot wound 261
Hill, Mr. on ptyalism 275
Hospitals, royal, extract from Holinshed on the establishment of 306
Hogben, Mr. prospectus of a treatise on midwifery 346
Hastings, Mr. on blisters 453
Harvey, Dr. a practitioner in midwifery, authorities for 470
Hydatids, natural history of, by Dr. Monro 510
Island, new volcanic, account of 80
Irvine, Dr. on the diseases of Sicily 65
Icelanders, on their diseases, by Dr. H. Holland 118
———, their diet 119
Inoculation, variolous, after vaccination 164
Intelligence, medical and philosophical 73, 168, 256, 338, 427, 518
Irvine, Dr. his death at Malta, and proposed account of Sicily 172

Ink, writing, M. Tarry's report of a memoir on 224
Iris, on the inflammation of 232
Intellection, what 441
Insanity, Mr. Crowther on 513
——, effects of local diseases on 514
Jugular vein, internal obliteration of 163
Jukes, Mr. Alfred, instrument for removing cartilage 372
Knowles, Mr. reply to a Norfolk practitioner 42
——, observations on his reply 189
——, Mr. reply to an Essex practitioner 370
Kennon, Mrs. the midwife, anecdotes of 380
Light, its agency in promoting the coloration of plants 18
Linnæus, his Lachesis Lapponica, observations on 21
Lectures announced.—*Anatomy*, Brooks, Armiger, 79, 80. *Midwifery*, Dr. Clough, Dr. Clarke, Mr. Hopkins, Dr. Ramsbotham, Dr. Merriman, 79, 80. *Theory and Practice of Medicine*, Dr. Clutterbuck, Dr. Adams, Dr. Buxton, and at the Middlesex hospital, 79, 80. On *Electricity*, at the Russel Institution, by Mr. Singer, 79, 431.—By Dr. Reid, Hooper and Agar, Moor (dentist), Singer, and Lydiat, 172, 257. By Mr. Stevenson, Dr. Adams, Drs. Hooper and Agar, Dr. Squire, Dr. G. Pearson, 523. Mr. T. Thomson, on *Botany*, 524.
Liver, case of abscess in 96
Lead, acetite of, given for hæmorrhage, produces paralysis 100
Latin, Sydenham's knowledge of 117
Leprosy in Iceland 120
Leucorrhœa 142, 148
Lues venerea 142
Lardner, on obliteration of the internal jugular 163
Legallois, M. on the power of animals to live without respiration 375
Language, medical remarks on 496, 513
Lambe, Dr. paper on arsenic, read to the Royal Society 518
Leslie, Prof. experiments on heat repeated in France 520
Marsh miasmata, observations on 37
Milward, Dr. memoirs of 49
Medical and philosophical intelligence 73, 168, 256, 338, 427, 518
Medicine, half-yearly view of 2
Mermaid, relation of one seen on the coast of Scotland 76
Meteorological table 87, 172, 263, 351, 439, 527
Midwifery, early practitioners of, in England 117
Measles inoculated 118
Meyer, Dr. German syphilitic physician 137
Masturbation, symptoms and effects of 139, 140
Montain, Dr. on apoplexy 151
Mitchell, Dr. of New York, his own case of croup 213
Muter, Mr. on cataract and artificial pupil 318
Medical Society of London, its annual meeting and oration 338
Museum of nat. hist. Mr. Bullock's 341
Mandrake, correction of a serious mistake respecting 348
Membrane, adventitious in croup, what 354, 361
——, mucous and serous, observations on 357
——, inflammation of ib.
Mermaid of Haerlem 382
Muscicapæ, certain vegetable, observations on, by Barton 388
Menu and the Caballa notions of the soul 442
Medical practitioners, on the remuneration of 462
Naturalist's monthly report 85, 172, 264, 349
Nyctalopia tropical, observations on 165
Newton's return to nature 318
Opium, observations on its *modus operandi* 191, 504
Ophthalmia, on some of its important terminations 234
Opium, its use in acute rheumatism, 286; in dysentery 293
Pithed, the term explained 8
Parkinson, Mr. on the strata and fossil remains in the neighbourhood of London 27
Progress of medicine, half-yearly view of 2
Patents, new 77
Publications, notices of intended 78, 342, 344, 345, 346, 429
Pearson, Mr. J. experiments with essential oil of almonds 6
Physiognomy, medical, observations on 38
*Prunus spinosa*, flowers of, a cure for fistula in ano 75
Paxton, Dr. case of abscess of the liver 96
Physicians, when first visiting in carriages 115
—— and apothecaries in partnership 116
—— compelled to temporise 117
Psora, common in Iceland 122
Paralysis, case of, cured by cantharides 149

Portal de, and Pelltier, on medicinal preparations of gold 156
Population, comparative statement of, in England, &c. in 1801 and 1811 169
Puerperal fever, observations on by Dr. Bradley 183
*Polygala senega*, its use in croup 209, 211
Plague, Dr. Whyte's observations on 271
Ptyalism, Mr. Hill's observations on 275
Pupil artificial, remarks on 322
Pursh, Mr. prodromus *Floræ Borealis Americanæ* 344
————, on the *eupatorium perfoliatum* 449
Phythian, Mr. on medical electricity 451
Pigot, Dr. on purging in typhus 458
*Pantherion* in London Museum, what 518
Puberty, early instance of 522
Queries, on the genus euphorbium, some species of agarics, the ranunculus aquaticus, birds, and the caoutchoc 380
Quickening, facts and observations on 441
————, Denman's account of, 443. Professor Hamilton on, 444. Burns on, ibid. London Practice of Midwifery and Dr. Clarke, on, 445.
————, objections to the received opinion of 446
————, its real nature 447
Respiration, chemical theory of, defended, 9, nota
Rain, quantity of, vide Meteorological Tables 87
Ruspini's styptic given in hæmorrhage from the lungs without benefit 101
Rheumatism common in Iceland 123
Robertson, Mr. diseases of the generative system 139
Ramsbotham, Dr. case of sudden death in pregnancy, with dissection 179
Ring-worm, cure for 187
Rot in the Zetland sheep, account of 223
Rabies canina, its universal fatality 237
————, character of, in the dog 283
————, indelibly marked by nature 240
————, arises spontaneously in the dog 244
————, nature of its poison 246
————, aphorisms on 247
Rabid animal, treatment of its bite 248
————, consequences of its bite 253
Rheumatism, case of 287
Scarlatina anginosa, on the treatment of 35
Sensation, Smith's theory of 38
Spencer, Mr. on cynanche trachealis 45
Stones, meteoric 82
Society, Royal, meetings of 73, 168, 256, 340, 427, 518
Siberia, new, discoveries in 77
Smith, Dr. president of the Linn. Soc. his translation of the *Lachesis Lapponica* 21
Smith, Mr. observations on the species of scarlatina 102
*Scarlatina tonsillitis* described 103
———— *maligna* described 104
Scott, Mr. J. R. medical sketches taken in the R. N. 89, 189, 284, 472
————, on depletion by the lancet 89, 189, 284
Scarlatina, on the treatment of 109
Small-pox, Sophia Bartlet's case, with observations 110
Sight, short, new cause of, by Dr. Wells 114
Small-pox, extraordinary success in treating the natural 115
Scotland, state of surgery in 1585 116
Surgery, state of, in Scotland in 1585 116
Sydenham's knowledge of Latin 117
Small-pox, its ravages in Iceland 121
Syphilis unknown in Iceland 122
Strictures, observations on the treatment of 140, 147
Stomach, severe affection of, from the external application of muriate of mercury 167
Silver, vein of, discovered in Cornwall 169
Skiddaw, relative state of barom. and therm. at the foot and summit of 170
Sheep, the Zetland, nat. and med. history of 216
Scab in sheep, how first brought to Zetland 221
Swallows, their migration 271
Society for relief of widows of medical men 283
Stamina and pistilla, their importance in the fructification of plants 318
Saunders, the late Mr. his character 238
Society, philosophical, of London 340
Sea-water baths, extensive erection of, at London 342
—, phosphorescent appearance of 377
Salts, triple experiments on 406
Sims, Dr. death of 437
Soul, opinions of the antients respecting 441
Stomach, human, extraordinary capacity of 453

Strength of men and horses in moving machines, experiments to ascertain 486
Society, Kirwanian, of Dublin, institution of 519
Terms, new, observations on the employment of 15
Tobacco, its effects in a case of psora 45
Travers, Mr. on wounds of the intestines 55
Twin-cases, observations on 109
Treatises, popular, on diseases and medicines 116
*Trismus nascentum*, its frequency and fatality in Iceland 123
Titford, Dr. *Hortus Botanicus Americanus* 159
Trance, instance of an extraordinary, in India 227
Titford, Dr. observations on his *Hortus Botanicus Americanus* 260
Thompson, Dr. on abscess of the liver, and nature of the bile 280
Tobacco and snuff, manufacture of, in New Spain 311
Temple, Dr. his oration at Med. Soc. of Lond. 338
Transactions of the Imperial Institute of France—Anatomy, physiology, and zoology, 374. Medicine and surgery, 377. Veterinary art and agriculture, 379.
Typhus, on purging in, by Dr. Pigot 458
Vena saphena major, operation for varicose 29
Vaccination, its progress and effects 34
Venesection, observations on the utility of, in inflammation 90, 91
Visceral inflammation common in Iceland 122
Vaccination, introduction of, into Iceland 103
Urethra, on strictures in 140, 147, 148
Vaccine and chicken pox together in the same person 171
Vegetable regimen, arguments in favor of 314
———, effects of, in the author's family 315
Vaccination, reception of, by the Five Nations 383
Universities, German, some account of 385
Vegetable muscicapæ, observations on, by Prof. Barton 388
Vermes, intestinal, natural history of 512
Wax, a vegetable one, account of, by Mr. Brande 73
White, Mr. on the Malabar Cardamom 20
Wells, Dr. on the cause of shortness of sight, 114, nota
Whately, Mr. case of procidentia ani 269
Whyte, Dr. on the plague 271
Washbourne, Mr. case of Tic-doloureux 281
———, of cynanche trachealis 282
Water, remarks on Dr. Lambe's opinions on 313
Wind of a Ball, on its effects 323
———, John Bell's objections to its existence 326
———, similar to atmospherical electricity 326
Wound, lacerated, extraordinary recovery from 464
Willan, Dr. death of in the Island of Madeira 524
Zetland sheep, nat. and med. history of 216

### PLATES.

Adventitious Membrane found in the Trachea in Croup Page 197
Stomach of a Rabid Dog - - - - - - - 296
Instrument for removing Cartilage - - - - - 374
Signatures of the Five Nations - - - - - - 385

